# Checklist of flesh flies of Turkey (Diptera, Sarcophagidae)

**DOI:** 10.3897/zookeys.743.22022

**Published:** 2018-03-14

**Authors:** Yury Verves, Miroslav Barták, Štěpán Kubík

**Affiliations:** 1 Institute for Evolutionary Ecology, National Academy of Sciences of Ukraine, Academician Lebedev Str. 37 Kyiv, 03143, Ukraine; 2 Department of Zoology and Fisheries, Faculty of Agrobiology, Food and Natural Resources, Czech University of Life Sciences, Prague, Kamýcká 129, 165 00 Praha Suchdol, Czech Republic

**Keywords:** checklist, distribution, Sarcophagidae, Turkey

## Abstract

A checklist of 153 flesh fly species (Diptera, Sarcophagidae) recorded to date from Turkey is presented. Updating the list was necessary due to the numerous recent records. Records are listed according to provinces.

## Introduction

The first reviews of Turkish Sarcophagidae were compiled by Verves (1986a, b) and Kara and Pape (2002), and they listed a total of 85 and 81 species, respectively. However, the true number of Turkish sarcophagids may well range from 175 to 250 species. For a comprehensive list of papers dealing with Turkish flesh fly fauna, consult Verves et al. (2017) or citations below. The need to elaborate the checklist of Turkish sarcophagids became evident after the authors presented numerous new records for Turkey and individual Turkish provinces (Verves et al. 2017).

## Materials and methods

Subfamilies, genera, and species are arranged in the order of the catalogue of Verves (1986a) with subsequent additions (Povolný and Verves 1997; Verves 1986b, 2001a, b; Verves and Khrokalo 2006a, b, 2007, 2009, 2014a, b, 2015; Verves et al. 2015a, b; Xue and Verves 2009; Xue et al. 2011, 2015). We are aware of conflicts between this conception and recent molecular studies (e.g. Piwczyński et al. 2017; Buenaventura and Pape 2017), so we used more traditional approach.

Distributional data of sarcophagids in Turkey were compiled analysing all the available publications (see references below). General species distribution was derived mostly from Pape (1996); Pape et al. (2015); Povolný and Verves (1997); Verves and Khrokalo (2006a, b, 2009, 2015); Verves et al. (2015b); Xue et al. (2011, 2015). Additional sources were as follows: Pape et al. (2002), Abasa (1970); Abd-AlGalil and Zambare (2016); Abdul-Rassoul et al. (2004); Al-Ghzawi et al. (2009); Al-Mesbah et al. (2012); Aldrich (1916); Amoudi (1993); Arnaud (1975); Arthur and Coppel (1953); Baranov (1929, 1938, 1942); Barnett and Emms (1998); Beaver (1986); Bezzi (1913, 1915, 1921); Blackith et al. (1997); Boscarelli and Sandri 2016); Brèthes (1908); Brimley (1938); Brink (2009); Britton (1921); Britvec (2000); Bruce and Knipling (1936); Bryan (1926); Buckell and Spencer (1945); Byers (1962); Calero (1948); Carles-Tolrá and Rosado 2009); Chaiwong et al. (2009); Clausen (1978); Cockerell (1905); Coe (1960, 1962); Criddle (1927); Curran (1929); Davis (1971); Dear (1980); Demyanova et al. (2007); Diaz and Kaufman (2011); Disney (1973); El-Hawagry et al. (2016); Feng et al. (2012); Fetene and Worku (2009); Frost et al. (2010); Gatt and Ebejer (2014)); Goff (1991); Goff and Odom (1987); Grabovac and Pertič (2005); Grigorian (1988); Hardy (1980); Ho (1938); Hutson (1981); James (1947); Johnson (1912); Kano et al. (1999); Kehlmaier (1998); Khan and Khan (1984); Krishnamurti and Usman (1955); Krüger et al. (2003); Kurahashi and Chaiwong (2013); Kurahashi and Kakinuma (2015); Kurahashi and Leh (2007); Kurahashi and Tan (2009); Lehrer (1975, 1996, 1999a, 2000, 2003, 2006b, 2007, 2008b); Lehrer and Oprisan (2012); Lopes (1958, 1959, 1961, 1967); Ma et al. 2014); Magnarelli and Andreadis (1981); Martínez-Sánchez et al. (2000); Maurya et al. (2012); Meiklejohn (2012); Nandi (2002, 2005); Nash (2005); Nazni et al. (2007); Nishida (2008); Pai et al. (2014); Pakalniškis and Podénas (1992); Pape (1991, 2004); Pape et al. (2002); Parker (1914); Peris et al. (1999); Pohjoismäki and Kahanpää (2014); Popov (1959); Povolný (1978, 1987, 1992, 1999); Povolný and Hula (2004); Prado e Castro et al. (2010); Rebelo et al. (2014); Rees (1985); Reed (1974); Richet et al. (2011); Rohdendorf (1937, 1975); Rouse (1967); Salem (1935, 1946); Senior-White et al. (1940); Shazia et al. (2006); Shinonaga (2001, 2004); Shinonaga and Thinh (2003); Sijstermans (2012); Silahuddin et al. (2015); Sinha (2014); Sisojević and Čepelák (1998); Sjüstedt (1935); Soler-Cruz (2000); Spuris (1976); Strickland (1938); Strukan (1968, 1970); Sucharit et al. (1976); Sugiyama et al. (1987, 1988); Szpila (2010); Tan et al. (2010); Thinh (1988, 2004); Tschirnhaus et al. (2000); Tucker (1906); Udgaonkar et al. (2012); Vairo et al. (2011); Verves (1978, 2001b, 2003, 2007); Verves et al. (2015a); Verves and Khrokalo (2014a, b); Wang et al. (2009); Webster (1907); Wei (2007); Whitmore (2009, 2011); Whitmore et. al (2013); Williams (1956); Wobeser et al. (1981); Wyatt (1991); Wyatt and Falk (1995); Zaidi et al. (2011, 2016); Zerova et al. (2006)); Zhang D et al. (2016); Zhang B et al. (2010); Zhang Q et al. (2014); Zumpt (1972).

Abbreviations: Provinces are abbreviated as follows: Adana (AD), Adiyaman (ADI), Afyon (AF), Ağrı (AG), Amasya (AM), Ankara (AN), Antalya (ANT), Artvin (AR), Aydın (AY), Batman (BT), Bayburt (BY), Bolu (BO), Burdur (BU), Bursa (BS), Diyabakir (DB), Elazığ (EL), Çanakkale (CA), Denizli (DE), Düzce (DU), Edirne (ED), Erzincan (ER), Erzurum (ERZ), Eskişehir (ES), Gaziantep (GA), Hakkâri (HA), Hatay (HT), Iğdır (IG), Isparta (IP), Istanbul (IS), İzmir (IZ), Karaman (KM), Kars (KAR), Kayseri (KY), Kırıkkale (KI), Konya (KN), Manisa (MN), Mardin (MR), Mersin (ME), Muğla (MG), Nevşehir (NE), Samsun (SA), Şanlıurfa (SN), Tokat (TO), Trabzon (TB), Van (VA).

## Checklist

### Subfamily Macronychiinae Brauer & Bergenstamm, 1889

**1. *Macronychia* (s. str.) *lemariei* Jacentkovský, 1941**

**Distribution in Turkey**: (Koçak 2014; Koçak and Kemal 2012): **AN** (Koçak & Kemal 2009, 2013, 2015), **KI** (Koçak and Kemal 2009, 2013, 2015).

**Distribution**: Palaearctic.

**2. *Macronychia* (s. str.) *striginervis* (Zetterstedt, 1838)**

**Distribution in Turkey**: (Koçak 2014): **ERZ** (Koçak and Kemal 2015; Pekbey 2011; Pekbey and Hayat 2013b).

**Distribution**: Palaearctic-Afrotropical.

**3. *Macronychia* (*Moschusa*) *polyodon* (Meigen, 1824)**

**Distribution in Turkey**: (Koçak 2014): **ERZ** (Koçak and Kemal 2015; Pekbey 2011; Pekbey and Hayat 2013b).

**Distribution**: Palaearctic-Oriental.

### Subfamily Miltogramminae Brauer & Bergenstamm, 1889

**4. *Senotainia* (*Arrenopus*) *albifrons* (Rondani, 1859)**

**Distribution in Turkey**: **ANT** (Koçak and Kemal 2009, 2012, 2015; Kara and Pape 2002); **AY** (Verves et al. 2017), **MG** (Verves et al. 2017), **SA** (Verves et al. 2017).

**Distribution**: Palaearctic-Afrotropical-Oriental.

**5. *Senotainia* (s. str.) *conica* (Fallén, 1810)**

**Distribution in Turkey**: (Koçak 2014; Koçak and Kemal 2012): **KN** (Kara and Pape 2002; Koçak and Kemal 2009, 2013, 2015).

**Distribution**: Palaearctic.

**6. *Senotainia* (s. str.) *deserta* Rohdendorf, 1935**

**Distribution in Turkey**: (Zerova et al. 2006).

**Distribution**: Palaearctic.

**7. *Senotainia* (s. str.) *tricuspis* (Meigen, 1838)**

**Distribution in Turkey**: (Koçak 2014; Koçak and Kemal 2012): **ME** (Kara and Pape 2002; Koçak and Kemal 2009, 2013, 2015).

**Distribution**: Palaearctic.

**8. *Protomiltogramma fasciata* (Meigen, 1824)**

**Distribution in Turkey**: **ANT** (Kara and Pape 2002; Koçak and Kemal 2009, 2012, 2015).

**Distribution**: Palaearctic.

**9. *Pterella grisea* (Meigen, 1824)**

**Distribution in Turkey**: (Koçak 2014; Koçak and Kemal 2012): **KY** (Kara and Pape 2002; Koçak and Kemal 2009, 2013, 2015).

**Distribution**: Palaearctic.

**10. *Miltogramma aurifrons* Dufour, 1850**

**Distribution in Turkey**: **MG** (Verves et al. 2017).

**Distribution**: Mediterranean.

**11. *Miltogramma brevipila* Villeneuve, 1911**

**Distribution in Turkey**: **AY** (Verves et al. 2017).

**Distribution**: West-Central Palaearctic.

**12. *Miltogramma germari* Meigen, 1824**

**Distribution in Turkey**: (Kara and Pape 2002; Koçak 2014; Koçak and Kemal 2009, 2012, 2015).

**Distribution**: Palaearctic.

**13. *Miltogramma murina* Meigen, 1824**

**Distribution in Turkey**: (Povolný and Verves 1997): **MG** (Verves et al. 2017).

**Distribution**: West Palaearctic.

**14. *Miltogramma oestracea* (Fallén, 1820)**

**Distribution in Turkey**: (Koçak 2014; Koçak and Kemal 2012): **ANT** (Kara and Pape 2002; Koçak and Kemal 2009, 2013, 2015) **TO** (Kara and Pape 2002; Koçak and Kemal 2009, 2013, 2015).

**Distribution**: Palaearctic.

**15. *Miltogramma testaceifrons* (Roser, 1840)**

**Distribution in Turkey**: **MG** (Verves et al. 2017).

**Distribution**: Palaearctic-Oriental.

**16. *Miltogramma turkmenora* Rohdendorf, 1930**

**Distribution in Turkey**: **MG** (Verves et al. 2017).

**Distribution**: East Mediterranean-Mid-Asiatic.

**17. *Miltogrammidium taeniatum* (Meigen, 1824)**

**Distribution in Turkey**: (Koçak 2014; Koçak and Kemal 2012): **BU** (Kara and Pape 2002; Koçak and Kemal 2009, 2013, 2015), **TO** (Kara and Pape 2002; Koçak and Kemal 2009, 2013, 2015), **VA** (Kemal and Koçak 2017).

**Distribution**: Palaearctic-Oriental.

**18. *Craticulina barbifera* (Pandellé, 1895)**

**Distribution in Turkey**: **MG** (Verves et al. 2017).

**Distribution**: Mediterranean.

**19. *Craticulina tabaniformis* (Fabricius, 1805)**

**Distribution in Turkey**: (Koçak 2014; Koçak and Kemal 2012, Verves 1993a): **ANT** (Koçak and Kemal 2009, 2013, 2015).

**Distribution**: Palaearctic-Oriental.

**20. *Apodacra dispar* Villeneuve, 1916**

**Distribution in Turkey**: **AY** (Verves et al. 2017), **MG** (Verves et al. 2017).

**Distribution**: Palaearctic-Afrotropical.

**21. *Apodacra pulchra* Egger, 1861**

**Distribution in Turkey**: **KY** (Koçak and Kemal 2015; Verves et al. 2015b).

**Distribution**: Palaearctic.

**22. *Apodacra radchenkoi* Verves & Khrokalo, 2015**

**Distribution in Turkey**: **ANT** (Koçak and Kemal 2015; Verves et al. 2015b).

**Distribution**: Anatolian.

**23. *Apodacra seriemaculata* Macquart, 1854**

**Distribution in Turkey**: (Zerova et al. 2006): **ES** (Koçak and Kemal 2015).

**Distribution**: Palaearctic.

**24. *Sphecapatodes ornatus* Villeneuve, 1912**

**Distribution in Turkey**: **MG** (Verves et al. 2017).

**Distribution**: East Mediterranean-Mid-Asiatic.

**25. *Metopodia pilicornis* (Pandellé, 1895)**

**Distribution in Turkey**: (Kara and Pape 2002; Koçak 2014 – as *M. grisea*; Koçak and Kemal 2009 –as *M. pilicornis*; 2012, 2013, 2015 – as *M. grisea*): **MG** (Verves et al. 2017).

**Distribution**: Palaearctic.

**26. *Phylloteles pictipennis* Löw, 1844**

**Distribution in Turkey**: (Kara and Pape 2002; Koçak and Kemal 2009, 2012, 2013, 2015): **MG** (Löw 1844).

**Distribution**: Palaearctic-Oriental.

**27. *Amobia oculata* (Zetterstedt, 1844)**

**Distribution in Turkey**: (Koçak 2014; Koçak and Kemal 2012): **ANT** (Kara and Pape 2002; Koçak and Kemal 2009), **ERZ** (Koçak and Kemal 2015; Pekbey 2011; Pekbey and Hayat 2013b), **TO** (Kara and Pape 2002).

**Distribution**: Holarctic-Oriental.

**28. *Amobia signata* (Meigen, 1824)**

**Distribution in Turkey**: (Koçak 2014; Koçak and Kemal 2012): **ANT** (Kara and Pape 2002; Koçak and Kemal 2009, 2015), **ERZ** (Koçak and Kemal 2015; Pekbey 2011; Pekbey and Hayat 2013b), **MG** (Verves et al. 2017), **TO** (Kara and Pape 2002; Koçak and Kemal 2009, 2013, 2015).

**Distribution**: Palaearctic-Oriental.

**29. *Metopia argyrocephala* (Meigen, 1824)**

**Distribution in Turkey**: (Kara and Pape 2002; Koçak 2014; Koçak and Kemal 2009, 2012, 2015): **AY** (Verves et al. 2017), **MG** (Verves et al. 2017), **SA** (Verves et al. 2017).

**Distribution**: Palaearctic-Nearctic-Oriental-Neotropical.

**30. *Metopia grandii* Venturi, 1953**

**Distribution in Turkey**: **MG** (Verves et al. 2017).

**Distribution**: Palaearctic.

**31. Phrosinella (Asiometopia) kozlovi (Rohdendorf, 1925)**

**Distribution in Turkey**: **IG** (Verves and Khrokalo 2017)

**Distribution**: Palaearctic.

**32. *Phrosinella* (*Asiometopia*) *tadzhika* (Rohdendorf, 1935)**

**Distribution in Turkey**: (Koçak 2014; Koçak and Kemal 2012): **KN** (Kara and Pape 2002; Koçak and Kemal 2009, 2013, 2015), **TO** (Kara and Pape 2002).

**Distribution**: Palaearctic.

**33. *Phrosinella* (s. str.) *nasuta* (Meigen, 1824)**

**Distribution in Turkey**: (Koçak 2014; Koçak and Kemal 2012): **BU** (Kara and Pape 2002), **KN** (Kara and Pape 2002; Koçak and Kemal 2009, 2013, 2015), **MG** (Verves et al. 2017), **TO** (Kara and Pape 2002; Koçak and Kemal 2009, 2013, 2015).

**Distribution**: Palaearctic.

**34. *Sphenometopa* (*Euaraba*) *bifasciata* (Brauer & Bergenstamm, 1891)**

**Distribution in Turkey**: (Koçak 2014; Koçak and Kemal 2009, 2012): **BS** (Brauer et Bergenstamm 1891; Kara and Pape 2002; Koçak and Kemal 2015), **VA** (Koçak and Kemal 2013, 2015).

**Distribution**: East Mediterranean-Mid-Asiatic.

**35. *Sphenometopa* (*Euaraba*) *claripennis* (Villeneuve, 1933)**

**Distribution in Turkey**: (Koçak 2014; Koçak and Kemal 2012): **KN** (Kara and Pape 2002; Koçak and Kemal 2009, 2013, 2015), **NE** (Kara and Pape 2002; Koçak and Kemal 2009, 2013, 2015).

**Distribution**: East Mediterranean-Mid-Asiatic.

**36. *Sphenometopa* (*Euaraba*) *manni* (Brauer & Bergenstamm, 1891)**

**Distribution in Turkey**: (Koçak 2014; Kara and Pape 2002; Koçak 2014; Koçak and Kemal 2012, 2013, 2015).

**Distribution**: East Mediterranean.

**37. *Sphenometopa* (*Xantharaba*) *steini* (Schiner, 1862)**

**Distribution in Turkey**: (Koçak 2014; Koçak and Kemal 2012): **ANT** (Koçak and Kemal 2009, 2013, 2015), **MG** (Verves et al. 2017), **VA** (Kemal and Koçak 2017).

**Distribution**: East Mediterranean.

**38. *Paragusia elegantula* (Zetterstedt, 1844)**

**Distribution in Turkey**: (Kara and Pape 2002; Koçak 2014; Koçak and Kemal 2009, 2012, 2013, 2015): **MG** (Verves et al. 2017).

**Distribution**: Palaearctic.

**39. *Paragusia multipunctata* (Rondani, 1859)**

**Distribution in Turkey**: **MG** (Verves et al. 2017).

**Distribution**: Palaearctic-Afrotropical-Oriental.

**40. *Taxigramma heteroneura* (Meigen, 1830)**

**Distribution in Turkey**: (Kara and Pape 2002; Koçak 2014; Koçak and Kemal 2009, 2012, 2013, 2015): **AY** (Verves et al. 2017), **MG** (Verves et al. 2017).

**Distribution**: Holarctic-Oriental.

### Subfamily Paramacronychiinae Brauer & Bergenstamm, 1889

**41. *Nyctia halterata* (Panzer, 1798)**

**Distribution in Turkey**: (Koçak 2014; Koçakand& Kemal 2012): **AM** (Kara and Pape 2002; Koçak and Kemal 2009, 2013, 2015), **AY** (Verves et al. 2017), **MG** (Verves et al. 2017), **TO** (Kara and Pape 2002; Koçak and Kemal 2009, 2013, 2015).

**Distribution**: West Palaearctic.

**42. *Nyctia lugubris* (Macquart, 1843)**

**Distribution in Turkey**: (Koçak 2014; Koçak and Kemal 2012): **BU** (Kara and Pape 2002; Koçak and Kemal 2009, 2013, 2015), **MG** (Verves et al. 2017), **SA** (Kara and Pape 2002; Koçak and Kemal 2009, 2013, 2015).

**Distribution**: Submediterranean.

**43. *Agria affinis* (Fallén, 1817)**

**Distribution in Turkey**: (Koçak 2014; Koçak and Kemal 2012): **IP** (Koçak and Kemal 2009, 2013, 2015), **TO** (Kara and Pape 2002; Koçak and Kemal 2009, 2013, 2015).

**Distribution**: Palaearctic.

**44. *Agria mamillata* (Pandellé, 1896)**

**Distribution in Turkey**: (Koçak 2014; Koçak and Kemal 2012): **AN** (Kara and Pape 2002; Koçak and Kemal 2009, 2013, 2015), **ES** (Koçak and Kemal 2009, 2013, 2015).

**Distribution**: Palaearctic.

**45. *Angiometopa falleni* Pape, 1986**

**Distribution in Turkey**: (Koçak 2014): **ERZ** (Koçak and Kemal 2015; Pekbey 2011; Pekbey and Hayat 2013b).

**Distribution**: Palaearctic.

**46. *Brachicoma devia* (Fallén, 1820)**

**Distribution in Turkey**: (Koçak 2014): **ERZ** (Koçak and Kemal 2015; Pekbey 2011; Pekbey and Hayat 2013b).

**Distribution**: Holarctic-Oriental.

**47. *Sarcophila canaanita* Lehrer, 2007**

**Distribution in Turkey**: **ANT** (Verves et al. 2017), **AY** (Verves et al. 2017), **MG** (Verves et al. 2017).

**Distribution**: East Mediterranean.

**48. *Sarcophila latifrons* (Fallén, 1817)**

**Distribution in Turkey**: (Aksoy and Bahadıroğlu 2012; Koçak 2014; Koçak and Kemal 2012): **AD** (Kara and Pape 2002; Koçak and Kemal 2009, 2013, 2015), **ADI** (Gözüaçık and Mart 2009), **BT** (Gözüaçık and Mart 2009), **MR** (Gözüaçık and Mart 2009), **MG** (Verves et al. 2017), **SN** (Gözüaçık and Mart 2009; Kara and Pape 2002; Koçak and Kemal 2009, 2015).

**Distribution**: Palaearctic.

**49. *Sarcophila meridionalis* Rohdendorf & Verves, 1982**

**Distribution in Turkey**: (Koçak 2014; Koçak and Kemal 2012): **ER** (Koçak and Kemal 2015; Pekbey 2011; Pekbey and Hayat 2013b), **ERZ** (Koçak and Kemal 2013, 2015; Pekbey 2011; Pekbey and Hayat 2010, 2013b), **MG** (Verves et al. 2017).

**Distribution**: West-Central Palaearctic.

**50. *Wohlfahrtia bella* (Macquart, 1839)**

**Distribution in Turkey**: (Kara and Pape 2002; Koçak 2014; Koçak and Kemal 2009, 2012): **AG** (Koçak and Kemal 2015), **BY** (Koçak and Kemal 2015; Pekbey 2011; Pekbey and Hayat 2013b), **ERZ** (Koçak and Kemal 2015; Pekbey and Hayat 2010).

**Distribution**: Palaearctic-Afrotropical.

**51. *Wohlfahrtia magnifica* (Schiner, 1862)**

**Distribution in Turkey**: (Dinçer 1997; Dinçer et al. 2001; Koçak 2014; Koçak and Kemal 2009, 2012, 2013; Kurtpınar 1950): **AF** (Köse et al. 2013), **AN** (Açıkgöz et al. 2011; Çiftçioglu et al. 1997), **DB** (İpek and Şaki 2010), **EL** (Bayındır et al. 2010; Kökçam and Şaki 2005; Şaki 2004; Şaki and Özer 1999a, b), **ERZ** (Büyükkurt et al. 2008; Gümüşsoy et al. 2015; Kara and Arslan 2011; Koçak and Kemal 2015; Pekbey 2011; Pekbey and Hayat 2010, 2013b; Yazgi et al. 2009), **GA** (Özdemir et al. 2014), **HT** (Çevik et al. 2014), **IS** (Karaman et al. 2009), **IZ** (Ütük 2006), **KAR** (Akduman et al. 2010), **KI** (Aydenizöz and Dik 2008; Dik et al. 2012), **KN** (Dik et al. 2012; Övet et al. 2012; Özsoy et al. 2013), **SA** (Atmaca et al. 2009), **SN** (Sevgili et al. 2004), **TO** (Tuygun et al. 2009), **VA** (Kılınç et al. 2013; Yuca et al. 2005).

**Distribution**: Palaearctic-Afrotropical-Oriental.

**52. *Wohlfahrtia meigeni* (Schiner, 1862)**

**Distribution in Turkey**: (Koçak 2014; Koçak and Kemal 2012): **ERZ** (Koçak and Kemal 2015; Pekbey 2011; Pekbey and Hayat 2013b), **KY** (Hayat et al. 2008; Koçak and Kemal 2009, 2013, 2015).

**Distribution**: Holarctic-Oriental.

**53. *Wohlfahrtia nuba* (Wiedemann, 1830)**

**Distribution in Turkey**: **SN** (Sevgili et al. 2004).

**Distribution**: Palaearctic-Afrotropical-Oriental.

**54. *Wohlfahrtia trina* (Wiedemann, 1830)**

**Distribution in Turkey**: **SN** (Sevgili et al. 2004).

**Distribution**: Palaearctic-Afrotropical-Oriental.

### Subfamily Sarcophaginae Macquart, 1935

**55. *Agriella lindneri* (Rohdendorf, 1937)**

**Distribution in Turkey**: (Kara and Pape 2002; Koçak 2014; Koçak and Kemal 2009, 2012): **KN** (Kara and Pape 2002; Koçak and Kemal 2013, 2015; Rohdendorf 1937; Verves 1985).

**Distribution**: Anatolian.

**56. *Blaesoxipha batilligera* Séguy, 1941**

**Distribution in Turkey**: **BY** (Pekbey 2011; Pekbey and Hayat 2013c), **ERZ** (Pekbey 2011; Pekbey and Hayat 2013c), **KAR** (Pekbey and Eroğlu 2017).

**Distribution**: West Palaearctic.

**57. *Blaesoxipha calliste* Pape, 1994**

**Distribution in Turkey**: (Kara and Pape 2002; Koçak 2014; Koçak and Kemal 2009, 2012): **BS** (Kara and Pape 2002; Pape 1994), **IP** (Ebejer 2000).

**Distribution**: Anatolian.

**58. *Blaesoxipha cochlearis* (Pandellé, 1896)**

**Distribution in Turkey**: (Koçak 2014; Koçak and Kemal 2012): **AM** (Kara and Pape 2002; Koçak and Kemal 2009, 2013, 2015; Pekbey and Hayat 2013c), **AR** (Pekbey and Eroğlu 2017), **ERZ** (Pekbey 2011; Pekbey and Hayat 2010, 2013c). **KAR** (Pekbey and Eroğlu 2017).

**Distribution**: Palaearctic.

**59. *Blaesoxipha confusa* Villeneuve, 1912**

**Distribution in Turkey**: **SN** (Verves et al. 2017).

**Distribution**: West Palaearctic.

**60. *Blaesoxipha dupuisi* J. Léonide & J. –C. Léonide, 1973**

**Distribution in Turkey**: **BY** (Pekbey 2011; Pekbey and Hayat 2013c), **ERZ** (Pekbey 2011; Pekbey and Hayat 2013c).

**Distribution**: Palaearctic.

**61. *Blaesoxipha grylloctona* Löw, 1861**

Blaesoxipha (s. str.) laticornis: Pekbey 2011: 75, 76, 92.

**Distribution in Turkey**: (Koçak and Kemal 2015; Pape, 1994): **ER** (Pekbey and Hayat 2013c), **ERZ** (Pekbey and Hayat 2010, 2013c).

**Distribution**: Palaearctic.

**62. *Blaesoxipha laticornis* (Meigen, 1826)**

**Distribution in Turkey**: (Kara and Pape 2002; Koçak 2014; Koçak and Kemal 2009 – as *B. laticornis* & *B. plumicornis* (as 2 different species), 2012, 2013, 2015 – as *B. gladiatrix*), **BY** (Pekbey 2011 – as *B. plumicornis*; Pekbey and Hayat 2013c), **ER** (Pekbey 2011 – as *B. plumicornis*; Pekbey and Hayat 2013c), **ERZ** (Pekbey 2011 – as *B. plumicornis*; Pekbey and Hayat 2010, 2013c), **IG** (Pekbey and Eroğlu 2017), **KAR** (Pekbey and Eroğlu 2017).

**Distribution**: Palaearctic.

**63. *Blaesoxipha lautaretensis* Villeneuve, 1928**

**Distribution in Turkey**: (Kara and Pape 2002; Koçak 2014; Koçak and Kemal 2009, 2012, 2013, 2015).

**Distribution**: Palaearctic-Oriental.

**64. *Blaesoxipha litoralis* (Villeneuve, 1911)**

**Distribution in Turkey**: (Kara and Pape 2002; Koçak 2014; Koçak and Kemal 2009, 2012): **ER** (Koçak and Kemal 2013, 2015; Pekbey 2011; Pekbey and Hayat 2013c), **ERZ** (Koçak and Kemal 2013, 2015; Pekbey 2011; Pekbey and Hayat 2013c), **IG** (Pekbey and Eroğlu 2017), **KAR** (Pekbey and Eroğlu 2017).

**Distribution**: Palaearctic.

**65. *Blaesoxipha pygmaea* (Zetterstedt, 1844)**

**Distribution in Turkey**: (Kara and Pape 2002; Koçak 2014; Koçak and Kemal 2009; 2012): **AR** (Pekbey and Eroğlu 2017), **BY** (Koçak and Kemal 2013; Pekbey 2011; Pekbey and Hayat 2013c), **ER** (Koçak and Kemal 2013; Pekbey 2011; Pekbey and Hayat 2013c).

**Distribution**: Palaearctic-Afrotropical-Oriental.

**66. *Blaesoxipha redempta* (Pandellé, 1896)**

**Distribution in Turkey**: (Kara and Pape 2002; Koçak 2014; Koçak and Kemal 2009 – as *B. lapidosa*, see comment in Verves et al. 2017): **AR** (Pekbey and Eroğlu 2017), **BY** (Pekbey 2011; Pekbey and Hayat 2013c), **CA** (Calvert 1882), **ER** (Pekbey 2011 – as *B. lapidosa*; Pekbey and Hayat 2013c), **ERZ** (Pekbey 2011; Pekbey and Hayat 2010, 2013c), **IG** (Pekbey and Eroğlu 2017), **KAR** (Pekbey and Eroğlu 2017), **MG** (Verves et al. 2017), **SA** (Verves et al. 2017).

**Distribution**: Palaearctic-Afrotropical-Oriental-Australasian/Oceanian

**67. *Blaesoxipha rufipes* (Macquart, 1839)**

**Distribution in Turkey**: (Koçak 2014; Koçak and Kemal 2012 – as *B. filipjevi*): **ANT** (Kara and Pape 2002; Koçak and Kemal 2009 – as *B. rufipes*, 2013, 2015), **AR** (Pekbey and Eroğlu 2017), **BY** (Pekbey 2011; Pekbey and Hayat 2013c), **ER** (Pekbey 2011; Pekbey and Hayat 2013b), **ERZ** (Pekbey 2011; Pekbey and Hayat 2013c), **KAR** (Pekbey and Eroğlu 2017).

**Distribution**: Palaearctic-Afrotropical-Oriental-Australasian/Oceanian.

**68. *Blaesoxipha ungulata* (Pandellé, 1896)**

**Distribution in Turkey**: (Kara and Pape 2002; Koçak 2014; Koçak and Kemal 2009, 2012): **ERZ** (Koçak and Kemal 2013; Pekbey 2011; Pekbey and Hayat 2013c).

**Distribution**: West Palaearctic.

**69. *Blaesoxipha unicolor* (Villeneuve, 1912)**

**Distribution in Turkey**: **AR** (Pekbey and Eroğlu 2017), **ER** (Pekbey 2011; Pekbey and Hayat 2013c), **ERZ** (Pekbey 2011; Pekbey and Hayat 2013c), **KAR** (Pekbey and Eroğlu 2017).

**Distribution**: Palaearctic-Oriental.

**70. *Servaisia* (s. str.) *erythrura* (Meigen, 1826)**

**Distribution in Turkey**: (Koçak 2014; Koçak and Kemal 2012): **AR** (Pekbey and Eroğlu 2017), **ERZ** (Koçak and Kemal 2013, 2015; Pekbey 2011; Pekbey and Hayat 2010, 2013c).

**Distribution**: Palaearctic.

**71. *Servaisia* (s. str.) *rossica* (Villeneuve, 1912)**

**Distribution in Turkey**: **ERZ** (Pekbey 2011; Pekbey and Hayat 2013c).

**Distribution**: Palaearctic.

**72. *Servaisia* (s. str.) *rybaltschenkoi* (Verves, 1977)**

*Blaesoxipha ataturkia* Lehrer, 2008a: 25.

**Distribution in Turkey**: (Koçak 2014; Koçak and Kemal 2012): **HA** (Kemal and Koçak 2015; Koçak and Kemal 2013; Lehrer 2008a).

**Distribution**: Palaearctic.

**73. *Tephromyia grisea* (Meigen, 1826)**

**Distribution in Turkey**: **ERZ** (Pekbey 2011; Pekbey and Hayat 2013c).

**Distribution**: Palaearctic.

**74. *Ravinia pernix* (Harris, 1780)**

**Distribution in Turkey**: (Koçak 2014; Koçak and Kemal 2012): **AD** (Aslan and Çalışkan 2009; Kara and Pape 2002; Koçak and Kemal 2009, 2013, 2015), **AY** (Verves et al. 2017), **ER** (Pekbey 2011), **ERZ** (Pekbey 2011; Pekbey and Hayat 2010), **ES** (Aslan, 2006; Aslan and Çalışkan 2009), **IG** (Pekbey and Eroğlu 2017), **KAR** (Pekbey and Eroğlu 2017), **KY** (Hayat et al. 2008; Koçak and Kemal 2009, 2013, 2015), **KN** (Aslan 2006; Hayat et al. 2008; Kara and Pape 2002; Koçak and Kemal 2013, 2015), **ME** (Aslan 2006; Kara and Pape 2002; Koçak and Kemal 2009, 2013, 2015), **MG** (Verves et al. 2017), **SN** (Sevgili et al. 2004), **TO** (Aslan 2006; Hayat et al. 2008; Kara et Pape 2002; Koçak and Kemal 2009, 2013, 2015).

**Distribution**: Palaearctic-Afrotropical-Oriental.

**75. *Sarcotachinella sinuata* (Meigen 1826)**

**Distribution in Turkey**: (Koçak 2014; Koçak and Kemal 2012): **AM** (Kara and Pape 2002; Koçak and Kemal 2009, 2013, 2015), **ER** (Pekbey 2011), **ERZ** (Pekbey 2011), **KY** (Hayat et al. 2008; Koçak and Kemal 2009, 2013, 2015), **MG** (Verves et al. 2017), **TO** (Kara and Pape 2002; Koçak and Kemal 2009, 2013, 2015).

**Distribution**: Holarctic.

**76. *Helicophagella* (s. str.) *bellae* (Lehrer, 2000)**.

**Distribution in Turkey**: (Koçak 2014; Koçak and Kemal 2012): **ANT** (Kara and Pape 2002; Koçak and Kemal 2009, 2013, 2015), **BU** (Kara and Pape 2002; Koçak and Kemal 2009, 2013, 2015), **KM** (Kara and Pape 2002; Koçak and Kemal 2009, 2013, 2015); **MG** (Verves et al. 2017).

**Distribution**: East Mediterranean.

**77. *Helicophagella* (s. str.) *crassimargo* (Pandellé, 1896)**

**Distribution in Turkey**: (Koçak 2014; Koçak and Kemal 2012): **AM** (Kara and Pape 2002; Koçak and Kemal 2009, 2013, 2015), **AY** (Verves et al. 2017), **BY** (Pekbey 2011), **ER** (Pekbey 2011), **ERZ** (Pekbey 2011; Pekbey and Hayat 2010), **KY** (Hayat et al. 2008; Koçak and Kemal 2009, 2013, 2015), **MG** (Verves et al. 2017), **TO** (Kara and Pape 2002; Koçak and Kemal 2009, 2013, 2015).

**Distribution**: Palaearctic.

**78. *Helicophagella* (s. str.) *novella* (Baranov, 1929)**

**Distribution in Turkey**: **MG** (Verves et al. 2017), **SA** (Verves et al. 2017).

**Distribution**: West Palaearctic.

**79. *Helicophagella* (s. str.) *noverca* (Rondani, 1860)**

**Distribution in Turkey**: **ES** (Aslan, 2006; Aslan and Çalışkan 2009), **SA** (Verves et al. 2017).

**Distribution**: West Palaearctic.

**80. *Helicophagella* (s. str.) *novercoides* (Böttcher, 1913)**

**Distribution in Turkey**: (Koçak 2014; Koçak and Kemal 2012): **ANT** (Kara and Pape 2002; Koçak and Kemal 2009, 2013, 2015), **ERZ** (Pekbey 2011), **MG** (Verves et al. 2017).

**Distribution**: West Palaearctic.

**81. *Helicophagella* (*Parabellieria*) *dreyfusi* (Lehrer, 1994)**.

**Distribution in Turkey**: (Kara and Pape 2002; Koçak 2014; Koçak and Kemal 2012, 2013, 2015).

**Distribution**: Palaearctic-Oriental.

**82. *Helicophagella* (*Parabellieria*) *macrura* (Rohdendorf, 1937)**

**Distribution in Turkey**: **AY** (Verves et al. 2017).

**Distribution**: Palaearctic.

**83. *Helicophagella* (*Parabellieria*) *maculata* (Meigen, 1835)**

**Distribution in Turkey**: (Koçak 2014; Koçak and Kemal 2012, 2015).

**Distribution**: West Palaearctic.

**84. *Helicophagella* (*Parabellieria*) *melanura* (Meigen, 1926)**

**Distribution in Turkey**: (Kara and Pape 2002; Koçak 2014; Koçak and Kemal 2012): **ANT** (Verves et al. 2017), **AY** (Verves et al. 2017), **BY** (Pekbey 2011), **ER** (Pekbey 2011), **ERZ** (Pekbey 2011; Pekbey and Hayat 2010), **ES** (Aslan 2006; Aslan and Çalışkan 2009; Koçak and Kemal 2009, 2015), **IG** (Pekbey and Eroğlu 2017), **KAR** (Pekbey and Eroğlu 2017), **KY** (Hayat et al. 2008; Kara and Pape 2002; Koçak and Kemal 2009, 2015), **MG** (Verves et al. 2017), **SA** (Verves et al. 2017), **SN** (Hayat et al. 2008; Koçak and Kemal 2009, 2015), **TO** (Aslan 2006; Kara and Pape 2002).

**Distribution**: Palaearctic-Nearctic-Afrotropical-Oriental.

**85. *Helicophagella* (*Parabellieria*) *pachyura* (Rohdendorf, 1937)**

**Distribution in Turkey**: (Koçak, 2014): **BY** (Pekbey 2011), **ER** (Pekbey 2011), **ERZ** (Koçak and Kemal 2012, 2013, 2015; Pekbey 2011; Pekbey and Hayat 2010), **IG** (Pekbey and Eroğlu 2017), **KAR** (Pekbey and Eroğlu 2017).

**Distribution**: Mid-Eastern.

**86. *Phytosarcophaga* (s. str.) *destructor* (Malloch, 1929)**

**Distribution in Turkey**: (Koçak 2014; Koçak and Kemal 2012): **AD** (Kara and Pape 2002), **AY** (Verves et al. 2017), **MN** (Kara and Pape 2002; Koçak and Kemal 2009, 2013, 2015), **ME** (Kara and Pape 2002; Koçak and Kemal 2009, 2013, 2015).

**Distribution**: West Palaearctic-Afrotropical.

**87. *Artamonoviella monspellensia* (Böttcher, 1913)**

**Distribution in Turkey**: **BY** (Pekbey and Hayat 2013a), **ER** (Pekbey and Hayat 2013a), **ERZ** (Pekbey 2011; Pekbey and Hayat 2013a).

**Distribution**: Mediterranean.

**88. *Disacachaeta cucullans* (Pandellé, 1896)**

**Distribution in Turkey**: (Koçak 2014; Koçak and Kemal 2013, “Anatolia”: Rohdendorf 1937): **ER** (Pekbey 2011; Pekbey and Hayat 2011, 2013a), **ERZ** (Pekbey 2011; Pekbey and Hayat 2011, 2013a) **IG** (Pekbey and Eroğlu 2017), **KAR** (Pekbey and Eroğlu 2017).

**Distribution**: West Palaearctic.

**89. *Heteronychia* (*Boettcherella*) *helenae* (Trofimov, 1948)**

**Distribution in Turkey**: (Koçak 2014; Koçak and Kemal 2012): **ANT** (Verves and Khrokalo 2015), **AR** (Pekbey and Eroğlu 2017), **ERZ** (Pekbey 2011; Pekbey and Hayat 2011, 2013a), **IG** (Pekbey and Eroğlu 2017), **IZ** (Koçak and Kemal 2015; Whitmore 2011), **KAR** (Pekbey and Eroğlu 2017), **MG** (Verves et al. 2017).

**Distribution**: West-Central Palaearctic.

**90. *Heteronychia* (*Boettcherella*) *mutila* (Villeneuve, 1912)**

**Distribution in Turkey**: (Koçak 2014): **KN** (Kara and Pape 2002; Koçak and Kemal 2009, 2012, 2015).

**Distribution**: West Palaearctic.

**91. *Heteronychia* (*Boettcherella*) *setinervis* (Rondani, 1860)**

**Distribution in Turkey**: (Kara and Pape 2002; Koçak 2014; Koçak and Kemal 2012): **ANT** (Verves and Khrokalo 2015), **DE** (Koçak and Kemal 2015; Lehrer 1977), **ERZ** (Pekbey 2011; Pekbey and Hayat 2011), **GA** (Whitmore 2010), **HT** (Koçak and Kemal 2015; Whitmore 2010), **IG** (Pekbey and Eroğlu 2017), **KAR** (Pekbey and Eroğlu 2017), **KN** (Verves and Khrokalo 2015), **KY** (Hayat et al. 2008; Koçak and Kemal 2009, 2015), **ME** (Koçak and Kemal 2015; Verves and Khrokalo 2015; Whitmore 2010), **MG** (Verves et al. 2017), **SN** (Koçak and Kemal 2015), **TO** (Koçak and Kemal 2013, 2015; Whitmore 2010).

**Distribution**: West-Central Palaearctic.

**92. *Heteronychia* (*Ctenodasypygia*) *minima* (Rondani, 1862)**

**Distribution in Turkey**: (Kara and Pape 2002; Koçak 2014; Koçak and Kemal 2009, 2012): **ANT** (Verves and Khrokalo 2015), **AY** (Koçak and Kemal 2013, 2015) **GA** (Lehrer 1976b), **IZ** (Koçak and Kemal 2013, 2015, Whitmore 2011), **MG** (Verves et al. 2017), **SN** (Sevgilli et al. 2004).

**Distribution**: West Palaearctic.

**93. *Heteronychia* (*Ctenodasypygia*) *siciliensis* (Böttcher, 1913)**

**Distribution in Turkey**: (Koçak 2014; Koçak and Kemal 2012, 2013): **ANT** (Verves and Khrokalo 2015), **AY** (Lehrer 1976b), **IZ** (Koçak and Kemal 2015; Whitmore 2011), **SN** (Verves et al. 2017)

**Distribution**: Submediterranean.

**94. *Heteronychia* (*Ctenodasypygia*) *thirionae* (Lehrer, 1976)**

**Distribution in Turkey**: (Kara and Pape 2002; Koçak 2014; Koçak and Kemal 2009, 2012): **BS** (Koçak and Kemal 2013, 2015; Lehrer 1976a).

**Distribution**: Mediterranean.

**95. *Heteronychia* (s. str.) *anatolica* (Whitmore, 2011)**

**Distribution in Turkey**: (Koçak 2014; Koçak and Kemal 2012): **AN** (Koçak and Kemal 2013, 2015; Whitmore 2011), **ERZ** (Pekbey 2011; Pekbey and Hayat 2011), **NE** (Koçak and Kemal 2013, 2015; Whitmore 2011).

**Distribution**: Anatolian.

**96. *Heteronychia* (s. str.) *armeniaca* (Rohdendorf, 1937)**

**Distribution in Turkey**: (Koçak and Kemal 2015): **BY** (Pekbey and Hayat 2011, 2013a), **ER** (Pekbey and Hayat 2011, 2013a), **ERZ** (Pekbey 2011; Pekbey and Hayat 2011, 2013a).

**Distribution**: Mid-Eastern.

**97. *Heteronychia* (s. str.) *benaci* (Böttcher, 1913)**

**Distribution in Turkey**: (Koçak 2014 – as *Sarcophaga bezziana*): **KY** (Kara and Pape 2002 – as *Sarcophaga bezziana*; Koçak and Kemal 2009, 2015 – as *Sarcophaga bezziana*), **KN** (Kara and Pape 2002 – as *Sarcophaga bezziana*; Koçak and Kemal 2009, 2013, 2015 – as *Sarcophaga bezziana*).

**Distribution**: West Palaearctic.

**98. *Heteronychia* (s. str.) *bulgarica* (Enderlein, 1936)**

**Distribution in Turkey**: **AR** (Pekbey and Eroğlu 2017), **BY** (Pekbey 2011; Pekbey and Hayat 2011, 2013a), **ER** (Pekbey and Hayat 2011, 2013a), **ERZ** (Pekbey 2011; Pekbey and Hayat 2011, 2013a), **SA** (Verves et al. 2017).

**Distribution**: West Palaearctic.

**99. *Heteronychia* (s. str.) *clarahenae* Lehrer, 1999**

**Distribution in Turkey**: **BY** (Pekbey and Hayat 2011, 2013a), **ER** (Pekbey and Hayat 2011, 2013a), **ERZ** (Pekbey 2011; Pekbey and Hayat 2011, 2013a).

**Distribution**: Mid-Eastern.

**100. *Heteronychia* (s. str.) *consanguinea* (Rondani, 1860)**

**Distribution in Turkey**: (Koçak 2014; Koçak and Kemal 2012): **AD** (Hayat et al. 2008; Koçak and Kemal 2009, 2013, 2015), **ANT** (Kara and Pape 2002; Koçak and Kemal 2009, 2013, 2015).

**Distribution**: Palaearctic-Oriental.

**101. *Heteronychia* (s. str.) *haemorrhoa* (Meigen, 1826)**

**Distribution in Turkey**: (Kara and Pape 2002; Koçak 2014; Koçak and Kemal 2009, 2012, 2013, 2015).

**Distribution**: West Palaearctic.

**102. *Heteronychia* (s. str.) *haemorrhoides* (Böttcher, 1913)**

*Heteronychia wahisi* Lehrer, 1976b: 264.

**Distribution in Turkey**: (Kara and Pape 2002; Koçak 2014; Koçak and Kemal 2009, 2012): **AM** (Koçak and Kemal 2013, 2015; Whitmore 2010), **AY** (Verves et al. 2017), **ER** (Pekbey 2011; Pekbey and Hayat 2011, 2013a), **ERZ** (Pekbey 2011; Pekbey and Hayat 2011, 2013a), **HT** (Koçak and Kemal 2013, 2015; Lehrer 1976b), **MG** (Verves et al. 2017), **SA** (Verves et al. 2017), **TO** (Koçak and Kemal 2013, 2015; Whitmore 2010).

**Distribution**: Palaearctic.

**103. *Heteronychia* (s. str.) *infantilis* (Böttcher, 1913)**

**Distribution in Turkey**: (Koçak 2014; Koçak and Kemal 2012 – as *Sarcophaga bezziana*, 2013; 2015 – as *Sarcophaga infantilis*).

**Distribution**: West Palaearctic.

**104. *Heteronychia* (s. str.) *infixa* (Böttcher, 1913)**

**Distribution in Turkey**: **SA** (Verves et al. 2017).

**Distribution**: West Palaearctic.

**105. *Heteronychia* (s. str.) *kerteszi* (Villeneuve, 1912)**

**Distribution in Turkey**: (Koçak 2014; Koçak and Kemal 2012): **ANT** (Kara and Pape 2002; Koçak and Kemal 2009, 2013, 2015; Whitmore 2011), **IZ** (Koçak and Kemal 2013, 2015; Whitmore 2011), **MG** (Verves et al. 2017).

**Distribution**: East Mediterranean.

**106. *Heteronychia* (s. str.) *lacrymans* (Villeneuve, 1912)**

**Distribution in Turkey**: (Koçak 2014; Koçak and Kemal 2012): **AF** (Kara and Pape 2002 – as Sarcophaga (Heteronychia) zhelochovtzevi; Koçak and Kemal 2009 – as Sarcophaga (Heteronychia) zhelochovtzevi, 2013, 2015; Whitmore 2011), **AR** (Pekbey and Eroğlu 2017), **ER** (Pekbey 2011; Pekbey and Hayat 2011, 2013a), **ERZ** (Pekbey 2011; Pekbey and Hayat 2011, 2013a), **MG** (Verves et al. 2017).

**Distribution**: West Palaearctic.

**107. *Heteronychia* (s. str.) *pontica* (Rohdendorf, 1937)**

**Distribution in Turkey**: **SA** (Verves et al. 2017).

**Distribution**: East Mediterranean.

**108. *Heteronychia* (s. str.) *porrecta* (Böttcher, 1913)**

**Distribution in Turkey**: **SA** (Verves et al. 2017).

**Distribution**: West Palaearctic.

**109. *Heteronychia* (s. str.) *recta* (Rohdendorf, 1937)**

**Distribution in Turkey**: **BY** (Pekbey 2011; Pekbey and Hayat 2011, 2013a), **ER** (Pekbey and Hayat 2013a), **ERZ** (Pekbey 2011; Pekbey and Hayat 2013a).

**Distribution**: Mid-Eastern.

**110. *Heteronychia* (s. str.) *rondaniana* (Rohdendorf, 1937)**

**Distribution in Turkey**: (Koçak 2014): **AM** (Kara and Pape 2002; Koçak and Kemal 2009, 2012, 2013, 2015), **AR** (Pekbey and Eroğlu 2017), **BY** (Pekbey 2011; Pekbey and Hayat 2011, 2013a), **ER** (Pekbey 2011; Pekbey and Hayat 2011, 2013a), **ERZ** (Pekbey 2011; Pekbey and Hayat 2011, 2013a), **KY** (Hayat et al. 2008; Koçak and Kemal 2009, 2013, 2015), **TO** (Koçak and Kemal 2013, 2013).

**Distribution**: West Palaearctic.

**111. *Heteronychia* (s. str.) *schineri* (Bezzi, 1891)**

**Distribution in Turkey**: (Koçak 2014): **AM** (Kara and Pape 2002; Koçak and Kemal 2009, 2012, 2013, 2015), **BY** (Pekbey 2011; Pekbey and Hayat 2011, 2013a), **SA** (Verves et al. 2017), **TO** (Kara and Pape 2002; Koçak and Kemal 2009, 2013, 2015).

**Distribution**: West Palaearctic.

**112. *Heteronychia* (s. str.) *vagans* (Meigen, 1826)**

**Distribution in Turkey**: (Koçak 2014; Koçak and Kemal 2012): **AN** (Kara and Pape 2002; Koçak and Kemal 2009, 2013, 2015), **TO** (Kara and Pape 2002; Koçak and Kemal 2009, 2013, 2015).

**Distribution**: Palaearctic.

**113. *Heteronychia* (*Pandelleola*) *boettcheri* (Villeneuve, 1911)**

**Distribution in Turkey**: (Kara and Pape 2002 – as Sarcophaga (Heteronychia) taurica; Koçak 2014; Koçak and Kemal 2009 – as Sarcophaga (Heteronychia) taurica, 2012): **AM** (Koçak and Kemal 2013, 2015; Whitmore 2011), **ANT** (Verves and Khrokalo 2015), **AR** (Pekbey and Eroğlu 2017), **AY** (Verves et al. 2017), **BO** (Lehrer 1977 – as Heteronychia (Pandelleola) gaspari), **DU** (Koçak and Kemal 2015), **ER** (Pekbey 2011; Pekbey and Hayat 2011, 2013a), **ERZ** (Pekbey 2011; Pekbey and Hayat 2011, 2013a), **IG** (Pekbey and Eroğlu 2017), **ME** (Koçak and Kemal 2013, 2015; Whitmore 2011), **MG** (Verves et al. 2017), **SA** (Koçak and Kemal 2013, 2015; Whitmore 2011), **TO** (Koçak and Kemal 2013, 2015; Whitmore 2011).

**Distribution**: West Palaearctic.

**114. *Heteronychia* (*Pandelleola*) *filia* (Rondani, 1860)**

**Distribution in Turkey**: (Koçak 2014; Koçak and Kemal 2012): **AM** (Kara and Pape 2002; Koçak and Kemal 2009, 2013, 2015; Whitmore 2011), **ANT** (Kara and Pape 2002; Koçak and Kemal 2009, 2013, 2015; Verves and Khrokalo 2015; Whitmore 2011), **AR** (Pekbey and Eroğlu 2017), **AY** (Verves et al. 2017), **BY** (Pekbey 2011; Pekbey and Hayat 2011), **ER** (Pekbey 2011; Pekbey and Hayat 2011), **ERZ** (Pekbey 2011; Pekbey and Hayat 2011), **ES** (Aslan 2006; Aslan and Çalışkan 2009; Koçak and Kemal 2009, 2013, 2015), **IG** (Pekbey and Eroğlu 2017), **KAR** (Pekbey and Eroğlu 2017), **KY** (Hayat et al. 2008; Koçak and Kemal 2009, 2015), **MG** (Verves et al. 2017), **SA** (Kara and Pape 2002; Koçak and Kemal 2009, 2015, Verves et al. 2017), **TB** (Hayat et al. 2008; Koçak and Kemal 2009, 2013, 2015), **TO** (Kara and Pape 2002; Koçak and Kemal 2009, 2013, 2015; Whitmore 2011).

**Distribution**: West Palaearctic.

**115. *Heteronychia* (*Pandelleola*) *turana* (Rohdendorf, 1937)**

**Distribution in Turkey**: (Koçak 2014; Koçak and Kemal 2012; Pekbey et al. 2011a): **IG** (Koçak and Kemal 2013, 2015; Pekbey and Eroğlu 2017),

**Distribution**: East Mediterranean-Mid-Asiatic.

**116. *Karovia hirticrus* (Pandellé, 1896)**

**Distribution in Turkey**: **SA** (Verves et al. 2017).

**Distribution**: West Palaearctic.

**117. *Bellieriomima subulata* (Pandellé, 1896)**

**Distribution in Turkey**: **SA** (Verves et al. 2017).

**Distribution**: Palaearctic.

**118. *Krameromyia anaces* (Walker, 1849)**

**Distribution in Turkey**: (Koçak 2014; Koçak and Kemal 2012): **AM** (Kara and Pape 2002; Koçak and Kemal 2009, 2013, 2015), **ERZ** (Pekbey 2011), **KY** (Hayat et al. 2008; Koçak and Kemal 2009, 2013, 2015).

**Distribution**: West Palaearctic.

**119. *Myorhina* (s. str.) *lunigera* (Böttcher, 1914)**

**Distribution in Turkey**: **SA** (Verves et al. 2017).

**Distribution**: West Palaearctic.

**120. *Myorhina* (s. str.) *nigriventris* (Meigen, 1826)**

**Distribution in Turkey**: (Koçak 2014; Koçak and Kemal 2012): **AM** (Kara and Pape 2002; Koçak and Kemal 2009, 2013, 2015), **AR** (Pekbey and Eroğlu 2017), **BY** (Pekbey 2011), **ER** (Pekbey 2011), **ERZ** (Pekbey 2011; Pekbey and Hayat 2010), **MG** (Verves et al. 2017), **SA** (Verves et al. 2017), **TO** (Kara and Pape 2002; Koçak and Kemal 2009, 2013, 2015).

**Distribution**: Palaearctic.

**121. *Myorhina* (s. str.) *socrus* (Rondani, 1860)**

**Distribution in Turkey**: **MG** (Verves et al. 2017).

**Distribution**: West Palaearctic.

**122. *Myorhina* (s. str.) *soror* (Rondani, 1860)**

**Distribution in Turkey**: (Koçak 2014; Koçak and Kemal 2012): **AM** (Kara and Pape 2002; Koçak and Kemal 2009, 2013, 2015), **AR** (Pekbey and Eroğlu 2017), **AY** (Verves et al. 2017), **BY** (Pekbey 2011), **ER** (Pekbey 2011), **ERZ** (Pekbey 2011), **MG** (Verves et al. 2017), **SA** (Kara and Pape 2002; Koçak and Kemal 2009, 2013, 2015, Verves et al. 2017).

**Distribution**: West Palaearctic.

**123. *Pandelleana protuberans* (Pandellé, 1896)**

**Distribution in Turkey**: (Koçak 2014; Koçak and Kemal 2012, 2013, 2015, “Anatolia”: Rohdendorf 1937): **ANT** (Verves et al. 2017), **ERZ** (Pekbey 2011), **ES** (Aslan, 2006; Aslan and Çalışkan 2009).

**Distribution**: Palaearctic.

**124. *Pandelleana tahtaliana* Lehrer, 2004**

**Distribution in Turkey**: **KN** (Lehrer 2004), **KY** (Lehrer 2004), **MG** (Verves et al. 2017).

**Distribution**: Anatolian.

**125. *Pseudothyrsocnema spinosa* (Villeneuve, 1912)**

**Distribution in Turkey**: (Koçak 2014; Koçak and Kemal 2012): **AD** (Kara and Pape 2002; Koçak and Kemal 2009, 2013, 2015), **AY** (Verves et al. 2017), **MG** (Verves et al. 2017).

**Distribution**: West Palaearctic.

**126. *Sarina sexpunctata* (Fabricius, 1805)**

**Distribution in Turkey**: (Koçak 2014; Koçak and Kemal 2012): **BY** (Pekbey 2011), **ERZ** (Pekbey 2011), **SA** (Verves et al. 2017), **TO** (Kara and Pape 2002; Koçak and Kemal 2009, 2013, 2015).

**Distribution**: Palaearctic.

**127. *Thyrsocnema incisilobata* (Pandellé, 1896)**

**Distribution in Turkey**: (Koçak 2014; Koçak and Kemal 2012): **AM** (Kara and Pape 2002; Koçak and Kemal 2009, 2013, 2015), **ERZ** (Pekbey 2011), **IG** (Pekbey and Eroğlu 2017), **KAR** (Pekbey and Eroğlu 2017), **MG** (Verves et al. 2017), **SA** (Verves et al. 2017), **TO** (Kara and Pape 2002; Koçak and Kemal 2009, 2013, 2015).

**Distribution**: Palaearctic.

**128. *Bercaea africa* (Wiedemann, 1824)**

**Distribution in Turkey**: (Kara and Pape 2002; Koçak 2014; Koçak and Kemal 2012 – both Sarcophaga (Bercaea) africa and Sarcophaga (Bercaea) cruentata)): **BT** (Koçak and Kemal 2013, 2015 – both Sarcophaga (Bercaea) africa and Sarcophaga (Bercaea) cruentata)), **BY** (Pekbey 2011), **DB** (İpek et al. 2009, – as *Sarcophaga haemorrhoidalis*), **ED** (Çoban and Beyarslan 2013), **EL** (Şaki and Özer 1999a, b – as *Sarcophaga haemorrhoidalis*), **ERZ** (Pekbey 2011; Pekbey and Hayat 2010), **ES** (Aslan 2006; Aslan and Çalışkan 2009; Koçak and Kemal 2009, 2013, 2015 – both Sarcophaga (Bercaea) africa and Sarcophaga (Bercaea) cruentata), **KAR** (Hayat et al. 2008; Koçak and Kemal 2009, 2013, 2015 – both Sarcophaga (Bercaea) africa and Sarcophaga (Bercaea) cruentata), **KI** (Dik et al. 2012 – as *Sarcophaga haemorrhoidalis*), **KN** (Dik et al. 2012 – as *Sarcophaga haemorrhoidalis*), **ME** (Aslan 2006; Kara and Pape 2002), **MG** (Verves et al. 2017), **SN** (Sevgili et al. 2004), **TO** (Aslan 2006; Kara and Pape 2002), **VA** (Koçak and Kemal 2015 – both Sarcophaga (Bercaea) africa and Sarcophaga (Bercaea) cruentata; Özdal and Değer 2005 – as *Sarcophaga haemorrhoidalis*).

**Distribution**: Cosmopolitan.

**129. *Liopygia* (*Engelisca*) *surcoufi* (Villeneuve, 1913)**

**Distribution in Turkey**: **SA** (Verves et al. 2017).

**Distribution**: Mediterranean.

**130. *Liopygia* (*Jantia*) *crassipalpis* (Macquart, 1839)**

**Distribution in Turkey**: (Kara and Pape 2002; Koçak 2014; Koçak and Kemal 2009, 2012, 2015): **ERZ** (Pekbey 2011; Pekbey and Hayat 2010), **ES** (Aslan 2006; Aslan and Çalışkan 2009), **SN** (Sevgili et al. 2004), **TO** (Aslan 2006; Aslan and Çalışkan 2009).

**Distribution**: Cosmopolitan.

**131. *Liopygia* (*Thomsonea*) *argyrostoma* (Robineau –Desvoidy, 1830)**

**Distribution in Turkey**: (Kara and Pape 2002; Koçak 2014; Koçak and Kemal 2012, 2015): **BY** (Pekbey 2011), **DB** (İpek et al. 2009, 2011), **ER** (Pekbey 2011), **ERZ** (Pekbey 2011; Pekbey and Hayat, 2010), **HT** (Koçak and Kemal 2009, 2013, 2015), **IG** (Pekbey and Eroğlu 2017), **IP** (Hayat et al. 2008; Koçak and Kemal 2009, 2013, 2015), **ME** (Hayat et al. 2008), **SN** (Sevgili et al. 2004).

**Distribution**: Cosmopolitan.

**132. *Liopygia* (*Varirosellea*) *uliginosa* (Kramer, 1908)**

**Distribution in Turkey**: **BY** (Pekbey 2011), **ERZ** (Pekbey 2011).

**Distribution**: Holarctic.

**133. *Liosarcophaga* (*Curranea*) *tibialis* (Macquart, 1851)**

**Distribution in Turkey**: **AN** (Açıkgöz et al. 2011), **AY** (Verves et al. 2017), **MG** (Verves et al. 2017), **SA** (Verves et al. 2017), **SN** (Sevgili et al. 2004).

**Distribution**: Palaearctic-Afrotropical-Oriental-Australasian/Oceanian.

**134. Liosarcophaga (s. str.) bartaki Verves, Radchenko & Khrokalo, 2017**

**Distribution in Turkey: AY** (Verves et al. 2017), **MG** (Verves et al. 2017), **SA** (Verves et al. 2017).

**Distribution**: Anatolian.

**135. Liosarcophaga (s. str.) dux (Thomson, 1869)**

**Distribution in Turkey**: **AN** (Açıkgöz et al. 2011), **SN** (Sevgili et al. 2004).

**Distribution**: Palaearctic-Afrotropical-Oriental-Australasian/Oceanian.

**136. *Liosarcophaga* (s. str.) *emdeni* (Rohdendorf, 1969)**

**Distribution in Turkey**: (Koçak 2014; Koçak and Kemal 2012): **AM** (Kara and Pape 2002; Koçak and Kemal 2009, 2013, 2015), **AR** (Pekbey and Eroğlu 2017), **ER** (Pekbey 2011), **ERZ** (Pekbey 2011), **SA** (Verves et al. 2017).

**Distribution**: Palaearctic.

**137. *Liosarcophaga* (s. str.) *fedtshenkoi* (Rohdendorf, 1969)**

**Distribution in Turkey**: **BY** (Pekbey 2011), **ERZ** (Pekbey 2011).

**Distribution**: Asian.

**138. *Liosarcophaga* (s. str.) *jacobsoni* (Rohdednorf, 1937)**

**Distribution in Turkey**: **ERZ** (Pekbey 2011; Pekbey and Hayat 2010), **ES** (Aslan 2006; Aslan and Çalýþkan 2009), **MG** (Verves et al. 2017).

**Distribution**: Palaearctic.

**139. *Liosarcophaga* (s. str.) *portschinskyi* (Rohdendorf, 1937)**

**Distribution in Turkey**: (Kara and Pape 2002; Koçak 2014; Koçak and Kemal 2009, 2012, 2015): **AM** (Koçak and Kemal 2013), **ERZ** (Pekbey 2011), **ES** (Aslan, 2006; Aslan and Çalışkan 2009), **SA** (Verves et al. 2017).

**Distribution**: Palaearctic-Oriental.

**140. *Liosarcophaga* (s. str.) *teretirostris* (Pandellé, 1896)**

**Distribution in Turkey**: **BY** (Pekbey 2011), **ERZ** (Pekbey 2011).

**Distribution**: West Palaearctic.

**141. *Liosarcophaga* (s. str.) *tuberosa* (Pandellé, 1896)**

**Distribution in Turkey**: **ER** (Pekbey 2011), **ERZ** (Pekbey 2011), **SN** (Sevgili et al. 2004).

**Distribution**: Palaearctic-Oriental.

**142. *Liosarcophaga* (*Pandelleisca*) *similissimilis* (Meade, 1876)**

**Distribution in Turkey**: (Koçak 2014; Koçak and Kemal 2012): **AY** (Verves et al. 2017), **MG** (Verves et al. 2017), **TB** (Kara and Pape 2002; Koçak and Kemal 2009, 2013, 2015).

**Distribution**: Palaearctic-Oriental.

**143. *Parasarcophaga* (s. str.) *albiceps* (Meigen, 1826)**

**Distribution in Turkey**: (Kara and Pape 2002; Koçak 2014; Koçak and Kemal 2009, 2012, 2015): **AR** (Pekbey and Eroğlu 2017), **ERZ** (Pekbey 2011), **SA** (Verves et al. 2017).

**Distribution**: Palaearctic-Oriental-Australasian/Oceanian.

**144. *Parasarcophaga* (s. str.) *hirtipes* (Wiedemann, 1830)**

**Distribution in Turkey**: (Kara and Pape 2002; Koçak 2014; Koçak and Kemal 2009, 2012, 2015): **SN** (Sevgili et al. 2004).

**Distribution**: Palaearctic-Afrotropical-Oriental-Australasian/Oceanian.

**145. *Robineauella* (s. str.) *caerulescens* (Zetterstedt, 1838)**

**Distribution in Turkey**: **ER** (Pekbey 2011).

**Distribution**: Holarctic-Oriental.

**146. *Rosellea aratrix* (Pandellé, 1896)**

**Distribution in Turkey**: (Koçak 2014; Koçak and Kemal 2012): **BU** (Kara and Pape 2002; Koçak and Kemal 2009, 2013, 2015), **SA** (Verves et al. 2017).

**Distribution**: Holarctic-Oriental.

**147. *Rosellea beckiana* Lehrer, 1996**

**Distribution in Turkey**: **AY** (Verves et al. 2017), **MG** (Verves et al. 2017).

**Distribution**: East Mediterranean.

**148. *Sarcophaga bergi* Rohdendorf, 1937**

**Distribution in Turkey**: (Kara and Pape 2002; Koçak 2014; Koçak and Kemal 2009; 2012): **ER** (Pekbey 2011, 2017), **ERZ** (Pekbey 2011, 2017), **ES** (Aslan and Çalışkan 2009; Pekbey 2017), **KAR** (Koçak and Kemal 2013, 2015; Rohdendorf 1937; Pekbey 2017; Pekbey and Eroğlu 2017).

**Distribution**: East Mediterranean.

**149. *Sarcophaga carnaria* (Linnaeus, 1758)**

*Sarcophaga schulzi*: Verves 1986: 188.

**Distribution in Turkey**: (Koçak, 2014; Koçak and Kemal 2015): **DB** (İpek et al. 2009), **SN** (Sevgili et al. 2004).

**Distribution**: Palaearctic.

**150. *Sarcophaga croatica* Baranov, 1941**

**Distribution in Turkey**: **ES** (Aslan and Çalışkan 2009; Pekbey 2017).

**Distribution**: Mediterranean.

**151. *Sarcophaga lehmanni* Müller, 1922**

**Distribution in Turkey**: (Kara and Pape 2002; Koçak 2014; Koçak and Kemal 2012 – as Sarcophaga (s. str.) lasiostyla): **AM** (Aslan 2006), **AR** (Pekbey and Eroğlu 2017), **AY** (Verves et al. 2017), **BY** (Pekbey 2011, 2017), **ER** (Pekbey 2011, 2017), **ERZ** (Pekbey 2011, 2017; Pekbey and Hayat, 2010), **ES** (Aslan, 2006; Aslan and Çalýþkan 2009; Koçak and Kemal 2009 – as Sarcophaga (s. str.) lasiostyla, 2013, 2015 – as Sarcophaga (s. str.) lasiostyla; Pekbey 2017), **HA** (Verves et al. 2017), **IG** (Hayat et al. 2008; Koçak and Kemal 2009 – as Sarcophaga (s. str.) lasiostyla, 2013, 2015 – as Sarcophaga (s. str.) lasiostyla; Pekbey 2017; Pekbey and Eroğlu 2017), **IZ** (Civelek and Tezcan 2005; Hayat et al. 2005; Koçak and Kemal 2009 – as Sarcophaga (s. str.) lasiostyla, 2013, 2015 – as Sarcophaga (s. str.) lasiostyla; Pekbey 2017), **KAR** (Hayat et al. 2008; Koçak and Kemal 2009, 2013, 2015 – as Sarcophaga (s. str.) lasiostyla; Pekbey 2017; Pekbey and Eroğlu 2017), **KY** (Hayat et al. 2008; Koçak and Kemal 2009 – as Sarcophaga (s. str.) lasiostyla, 2013, 2015 – as Sarcophaga (s. str.) lasiostyla; Pekbey 2017), **MLA** (Koçak and Kemal 2013), **MN** (Civelek and Tezcan 2005; Koçak and Kemal 2009 – as Sarcophaga (s. str.) lasiostyla, 2013, 2015 – as Sarcophaga (s. str.) lasiostyla), **MG** (Verves et al. 2017), **SA** (Verves et al. 2017).

**Distribution**: Palaearctic.

**152. *Sarcophaga trabzonensis* Pekbey, Hayat, Richet & Blackith, 2011**

**Distribution in Turkey**: **AR** (Pekbey, 2017; Pekbey et al. 2011b), **KAR** (Pekbey, 2017; Pekbey et al. 2011b), **TB** (Pekbey, 2017; Pekbey et al. 2011b).

**Distribution**: Anatolian.

**153. *Sarcophaga variegata* (Scopoli, 1763)**

**Distribution in Turkey**: (Kara and Pape 2002; Koçak 2014; Koçak and Kemal 2009, 2012 – as *Sarcophaga carnaria*; 2013 - as *Sarcophaga variegata*; 2015 - as *Sarcophaga carnaria*): **DB** (İpek et al. 2009), **EL** (Şaki and Özer 1999a, b), **SN** (Sevgili et al. 2004).

**Distribution**: Palaearctic.

## Discussion

**Figure 1.**
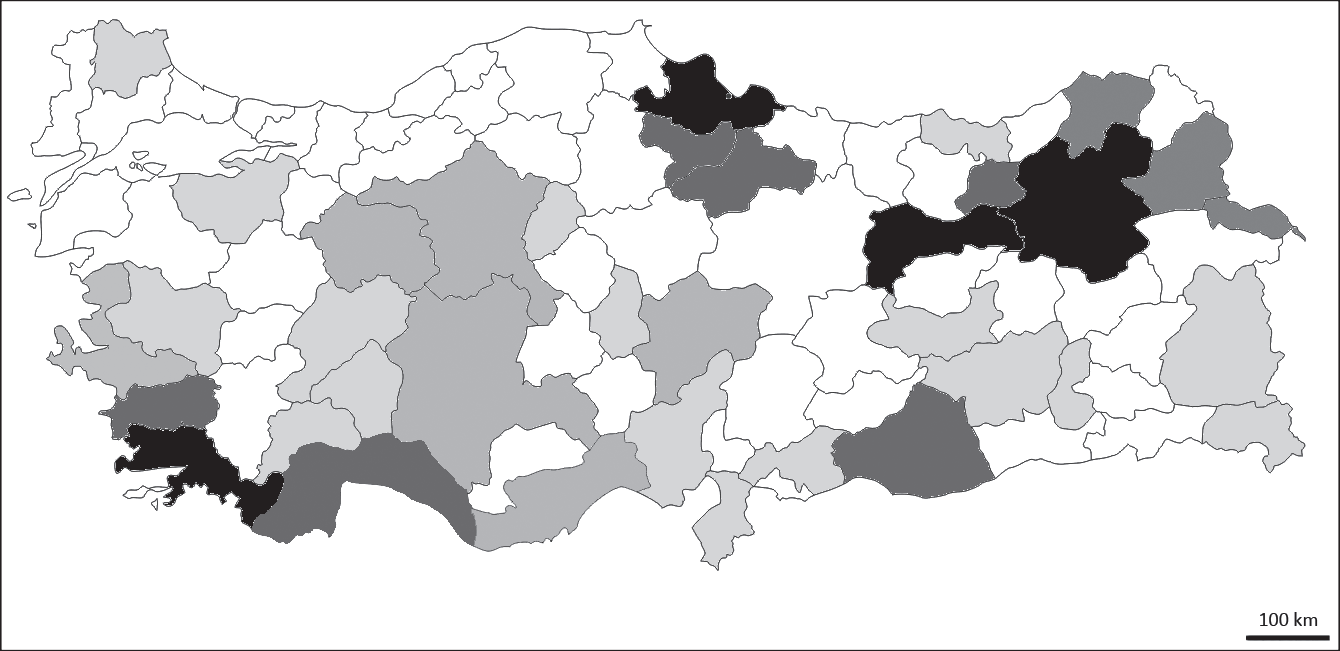
Level of flesh fly knowledge in individual Turkish provinces. Darker colour depicts higher number of species known (scale: 2–6, 7–14, 15–29, more than 30 species). Only provinces with more than two species are shaded.

Altogether 153 species of Sarcophagidae are listed from Turkey. The degree of study of Turkish sarcophagids is not high: we suppose that approximately 75 – 80 % of species is currently known from the country. The number of species known for each Turkish province is very different (Fig. 1), depending chiefly on areas of research of the most active researchers: 62 species are recorded from Erzurum, 54 from Muğla, 32 from Erzincan, 31 from Samsun, 27 from Bayburt, 25 from Tokat, 24 from Aydın, 23 from Antalya, 20 from Şanlıurfa, 19 from Artvin, 18 from and Kars, 16 from Amasya, and Iğdır, 14 from Eskişehir, and Kayseri, 12 from Konya, 7 from Ankara, İzmir, and Mersin, 5 from Adana, Burdur, Diyarbakır and Gaziantep, 4 from Hatay, 5 from Bursa, Elazığ, Isparta, Kırıkkale, Trabzon, and Van, 2 from Afyonkarahisar, Batman, Hakkâri, Manisa, and Nevşehir, 1 from Adiyaman, Ağrı, Bolu, Çanakkale, Denizli, Düzce, Edirne, Istanbul, Karaman, Malatya, and Mardin. No sarcophagids are known from the remaining provinces.

Many scientists think that Anatolia was an important Pleistocene glacial refugium. This fact, together with heterogeneous topography and geographical position of Anatolia at junction of three biodiversity hotpots: the Caucasus, Irano-Anatolian and Mediterranean (Gür 2016), resulted in very high animal diversity. This, alongside poor level of faunistic research, may explain recent increase in the number of known sarcophagids from 82 (last catalogue: Kara and Pape 2002) to 153 (present paper). Nevertheless, zoogeographical analysis of Turkish sarcophagid fauna is difficult because many species changed its range owing to human activities.

Altogether 24 species (ca. 16 %) are broadly distributed, known from at least three basic geographic realms. Out of them, three species are virtually cosmopolitan (*Bercaea africa, Liopygiaargyrostoma*, and *L. crassipalpis*). At least several species of this group were artificially disseminated by humans.

Moreover, 20 species (ca. 13 %) are widely distributed also in other zoogeographical regions: Oriental (14 species), Afrotropical (4 species), and Nearctic (2 species).

**Figure 2.**
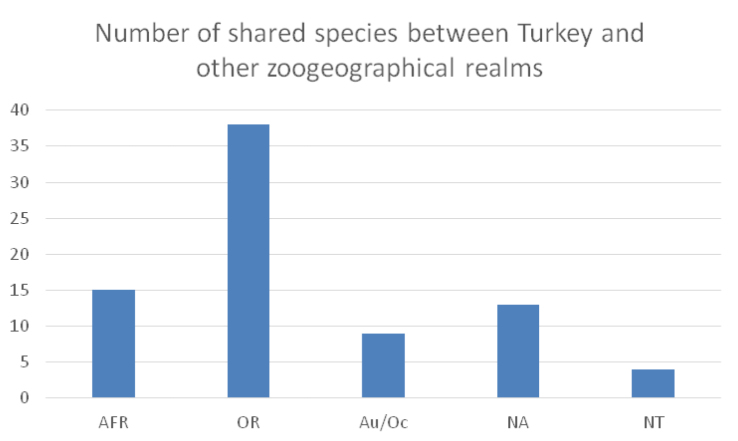
Numbers of shared species between Turkey and other zoogeographical realms.

The numbers of shared species between Turkey and other zoogeographical regions is depicted in Fig. 2.

Most species occur also in the Oriental (38 species), Afrotropical (15 species), Nearctic (13 species), Australian/Oceanian (9 species) and Neotropical (4 species) regions. Not surprisingly, most species known currently from Turkey are Palaearctic in distribution (43 species) or known from at least the western (30 species) or west – central parts (4 species), representing more than 50% of species. The dominance of Palaearctic elements in the Anatolian fauna has been well established (see e.g., Kosswig 1955).

The remaining species have several types of distribution covering the whole of the Mediterranean subregion (6 species) or only its eastern parts (8 species), while additional species penetrate from teh East Mediterranean to eastern countries of Middle East (4 species) or to the warmest parts of central Europe (2 species classified as submediterranean) or to central Asia (5 species). Altogether these species compose 16.33 % of total.

Faunistically significant are species up to now known only from Turkey, classified in the list above as Anatolian (*Apodacra radchenkoi, Agriella lindneri, Blaesoxipha calliste, Heteronychia anatolica, Liosarcophaga bartaki, Pandelleana tahtaliana, Sarcophaga trabzonensis* - 7 species, 4.58 %).
